# The prevalence of drug use and illicit trafficking: A descriptive cross sectional study of irregular migrant returnees in Nigeria

**DOI:** 10.1016/j.jmh.2021.100034

**Published:** 2021-01-26

**Authors:** Ikenna Daniel Molobe, Oluwakemi Ololade Odukoya

**Affiliations:** a Unified Initiative for a Drug Free Nigeria (UIDFN), Lagos, Nigeria; b Non-Communicable Disease Research Group, University of Lagos, Lagos, Nigeria; c Department of Community Health and Primary Care, College of Medicine, University of Lagos, Nigeria

**Keywords:** Irregular migration, Drug use, Drug trafficking, Mediterranean route, Libya, Nigerians

## Abstract

The study assessed the prevalence of drug use and drug trafficking among Nigerian returned migrants from Libyan detention centers in the transit or destination along the Mediterranean irregular migration route. This is a descriptive cross sectional study. The study population was restricted to migrants who returned from May 2017 and April 2018. A total of 382 (238 male and 144 female) were contacted and provided information for this study. These participants were recruited using judgemental and snowballing techniques. Both quantitative and qualitative methods of data collection were used. Results showed that 61.3% of the respondents used drugs during their migration. Drug use was predominant among those in the younger age group (26–30) accounting for 24.9%. The study revealed reasons for respondents’ drug use which were as a result of migration frustration and trauma, or compulsion. The findings on drug trafficking revealed that 15.7% of the respondents engaged in drug trafficking during their migration, and 28% of the respondents that trafficked drugs had previous experience of arrest or detention by law enforcement agent (in Libya). Findings from this study showed high prevalence of drug use among irregular migrants. Experience of migration frustration and trauma were among the factors that contribute to drug use among the migrant population. The study also discovered that some of the migrants who got into illicit drug trafficking were to raise money for survival while some were compelled into the business. The high prevalence of drug use among irregular migrants from findings draw attention to the important implications for public health and social security, while drug trafficking in existence among migrants calls for need of social reintegration.

## Highlights

• Drug use prevalence in irregular migration among study participants is 61.3%.
• Alcohol and marijuana is mostly the drug use among irregular migrants.
• Migration frustration and trauma are the major reason for drug use.
• Marijuana is most trafficked drug by irregular migrants.

## Introduction

Irregular migration, according to the International Organization for Migration (IOM), can be defined as entry into another country in contravention of immigration laws of that country. Therefore, irregular migrants include, among others, clandestine, illegal, unauthorized, unlawful, undocumented, aliens without residence status, illegalized people, non-compliant, and without documents (International Organization for Migration 2011).

According to IOM, many young people felt deceived about better options for life and work if they migrate (International Organization for Migration 2011). These young people often fall into wrong hands of organized recruiters who facilitate their movement outside the country (International Organization for Migration 2004). In reports, such as of the United Nations Office on Drugs and Crime (UNODC) and United Nations Interregional Crime and Justice Research Institute (UNCRI) on organized crime and irregular migration, human trafficking from Africa to Europe; some of these migrants are introduced into drug trafficking (UNCRI 2004; UNODC 2006). These migrants also face a lot of hardship and could be frustrated both on their way and to their final destination (UNODC 2011). Sometimes irregular migrants end up being stranded as many go into drug peddling, commercial sex work, street begging (UNCRI 2004; UNODC 2011; García and Laura, 2009), or even drug use as a result of psychological trauma (Borges et al., 2012). Some irregular migrants end up in prisons as a result of penalty against crimes committed and therefore constituting nuisance to the country where they migrated into (García and Laura, 2009).

As reported by the International Centre for Migration Policy Development (ICMPD), victims in most cases appear to be aware that they will be expected to work as drug peddlers at their destination, but it seems that they are often ignorant of the full implications (International Centre for Migration Policy Development 2004). Having committed themselves to drug traffickers or to the consortium that will control their activities, they become bond slaves without the right to opt out of their contract and are liable to be subjected to abuse of various sorts (International Centre for Migration Policy Development 2004).

Drug trafficking and drug use are present in transnational migration and among irregular migrants. As revealed in the study on Mexican immigration to the US and drug opportunities, irregular migrants are exposed to drugs and drug related behaviours and traditional approaches to address this are sparse (Borges et al., 2012). As a consequence, in such communities, a drug use culture is evolving; a culture that, if allowed to take root, will set the foundation for potentially serious future drug problems.

Studies, as described in the epidemiology of substance use among forced migrants by Horyniak, Melo, Farrell, Ojeda, and Strathdee; and the healthy immigrant effect: patterns and evidence from four countries by Kennedy, Kidd, McDonald, and Biddle; irregular migrants may be at risk for drug use for some reasons, including coping with traumatic experiences, pre-and post-migration stress, co-morbid mental health disorders, acculturation challenges, and social and economic inequality (Horyniak et al., 2016; Kennedy et al., 2015). Migrants, who also have passed through ill-treatment and abuse such as experienced by irregular migrants, can face marginalization, stigmatization and discrimination (Fozdar and Hartley, 2014; Capps et al., 2015), which is an important factor in determinants of health, and may contribute to feelings of stress and loss of hope which may, in turn, lead to drug use problem (Frohlich and Potvin, 2008; Steel et al., 2009). In addition, study by Anikeeva, Bi, Hiller, Ryan, Roder and Han; and Gushulak, Pottie, Roberts, Torres, and DesMeules, express that migrant health decreases over time to a range of factors, including reintegration challenges and barriers to health service use (Anikeeva et al., 2010; Gushulak et al., 2011; Gorman, 2014; Mojtabai et al., 2014).

Activities of drug trafficking networks and among irregular migrants constitute the extent of this drug problem. Drug traffickers and trafficking networks have found irregular migrant communities (migrants with false or no legal documents, smuggled migrants and trafficked persons) and their social networks very useful for their activities (Horyniak et al., 2016). Over the years, through these networks, countless migrants have made their way north to make a living (International Organization for Migration 2016a, 2016b). In specific terms, drug trafficking organizations contribute in facilitating the movement of illegal migrants into other countries using highly functional groundwork within the social network to achieve their purpose. At present, drugs flow through these networks, and an undetermined number of irregular migrants are being used for major distribution (Slack and Whiteford, 2010; Triandafyllidou et al., 2012). In the same communities, drug trafficking organizations and networks also target at-risk young people turning them into drug consumers as well as recruiting them to sell drugs, in some cases there are some migrants whose sole objective in migrating is to traffic in and sell drugs (García, 2007; Cook, 2007).

Nigeria presently faces a high rate of unemployment and conflicts, and youths are illegally migrating to other countries and involved in drug trafficking and drug use during their migration. Some of them were introduced by organized recruiters or migrant smugglers who facilitate their movement with drug trafficking within and outside Nigeria through their network system (Carling, 2014). Some of the irregular migrants that got stranded take to drug peddling, commercial sex work, street begging, among others, in order to survive. Some also fall victims of drug use as a result of psychological trauma (Borges et al., 2012), while some end up in prisons due to crime committed and thereafter repatriated to Nigeria after serving their time. On return to Nigeria, some of these migrants, particularly young people, are roaming the street without any meaningful achievement while some have re-migrated or suffered psychological trauma due to frustration. Some have also fall victim of drug use due to post migration trauma (International Centre for Migration Policy Development 2004; International Organization for Migration 2016a, 2016b). Europe has become one of the major continents with high rate of irregular migrants from Nigeria (UNODC 2011).

### Study objectives

1 To assess the prevalence of drug use among irregular migrants.
2 To examine the patterns of drug use among illegal migrants.
3 To investigate the nature of illicit trafficking among irregular migrants.

## Methods

### Description of the study setting

This study was carried out in Edo and Delta states of Nigeria among identified migrant returnees who were brought back from Libya by IOM and the Federal Government of Nigeria. These returnees were migrants of irregular migration who travelled by road and through the Sahara desert and intended to migrate to Europe through the Mediterranean Sea in search for greener pasture, and were stranded in Libya. According to the IOM Nigeria report, Edo states and Delta states account for communities of high emigration and high returns among the irregular migrant population in Nigeria. This informed the rationale behind chosen the two States for the study. Again, the states accounted for high potential migrants, and stranded and transiting migrants along Mediterranean Sea. Going by the IOM report of 2018; 6, 978 migrants returned as at February which extended to 7709 as at March and 8500 as at April; and Libya accounts for the highest population of migrants by countries; among these returnees (International Organization for Migration 2018).

### Type of study design

The study adopted the descriptive cross sectional survey design, as the research was only interested in determining the independent and dependent variable without manipulating any of them.

### Description of the study population

The population of the study was restricted to migrant returnees from Libya who returned between May 2017 and April 2018. The criterion for participation in the survey was that the respondents must have returned three months and have had reunion with their family before the interview or had undergone reintegration programme. The migrant returnees who have undergone reintegration programme are those that have received support from organizations upon their return aims to address their economic, social or psychosocial needs. The age consideration for the selection of respondents was within 18 – 50 years. This age bracket was the most predominant among the irregular migrants according to IOM. It was ensured that both genders were represented in the study.

### Sampling techniques

The study used judgemental and snowball sampling methods, and copies of questionnaire were properly administered to 382 participants (238 male and 144 female). In applying both sampling techniques, the study employed the use of a migration consultant, who is also a migrant returnee, working with the migrant returnees on reintegration and familiar with the community, who assisted in identifying and contacting the returnees (judgemental) and each identified migrant returnee assisted in recruiting other returnees (snowball) within their social network for this study. The sampling methods were adopted due to difficult to locate and hidden nature of the population. In addition to the administration of questionnaire, 4 focused group discussion (FGD) were conducted with 12 selected participants in each group. The FGD comprised 2 male and female sessions respectively. In total were 24 male and female FGD participants respectively. In-depth Interview (IDI) was conducted among 10 selected participants.

### Data collection instruments and procedure

The study employed both quantitative and qualitative methods. The research participants responded to In-depth Interview (IDI) and Focus Group Discussion (FGD) guide and interviewer administered structured questionnaire. The instruments were developed by the authors from the literature review and with consultations and guide from migration experts and professionals in the field of drugs and addiction. In addition, the IDI and FGDs were conducted with the participants to gain a deeper understanding of their experiences. Adopting this mixed method is important because it assists in developing robust explanations to the complexities of irregular migration, drug trafficking and drug use.

Through non-probabilistic purposeful sampling, 10 volunteer migrant returnees who had engaged in either drug trafficking or drug use were interviewed and it was ensured that important elements were selected to participate in the interview. Attention was focused on selecting informants from diverse groups that represent the community, such as irregular migrants, smuggled migrants, and trafficked persons. A total of 4 FGD sessions were conducted: 2 male and female sessions, respectively.

### Methods of data analysis

The quantitative data was analyzed using SPSS statistical tool (version 21.0). The data presented in this study is in accordance with the stated method, sample size, data collection instrument and method of data analysis. The quantitative analysis of findings and variables were based on descriptive statistics using frequency distribution and chi-square. The qualitative primary data obtained were manually analyzed by content analysis, reviews and discovery process, and by generating themes from FGD and in-depth interviews. Responses were coded into themes accordingly and categories abstracted into sub themes and data saturation was reached when no new information is discovered in data analysis. Comparison and verifying of conclusions were carried out and organized according to the set objectives of the study. Remarkable and very important quotes from the respondents were noted and referenced in the report.

### Ethical approval

Ethical approval for this study was received from the Health Research and Ethics Committee of Lagos University Teaching Hospital (LUTH). Confidentiality and anonymity was assured and informed oral consent obtained from all the respondents after explaining the purpose of the study in detail.

## Results

Table 1 shows the summary of the socio-demographic characteristics of the respondents. The male constitutes the highest number in the study participants, representing 238(62.3%). Most of the respondents 259(67.8%) only have secondary school level education. Marital status showed that most of the returnees 226(59.2%) were single. The age group of 26 – 30 years was the most dominant among the study population.

**Table 1:** Demographics characteristics of the respondents.

Characteristics	Frequency (*n* = 382)	Percentage (%)
**Gender**		
Male	238	62.3
Female	144	37.7
**Level of Education**		
No formal education	4	1.0
Primary Education	62	16.2
Secondary Education	259	67.8
Tertiary Education (NCE/OND)	38	9.9
Tertiary Education (B.sc/HND)	19	5.0
**Marital Status**		
Single	226	59.2
Married	107	28.0
Separated	36	9.4
Divorced	8	2.1
Widow/Widower	5	1.3
**Age (Years)**		
18–25	76	19.9
26–30	154	40.3
31–40	137	35.9
41–50	15	3.9
Mean	29.64±5.973	

### Country of migration

**Fig. 1:**
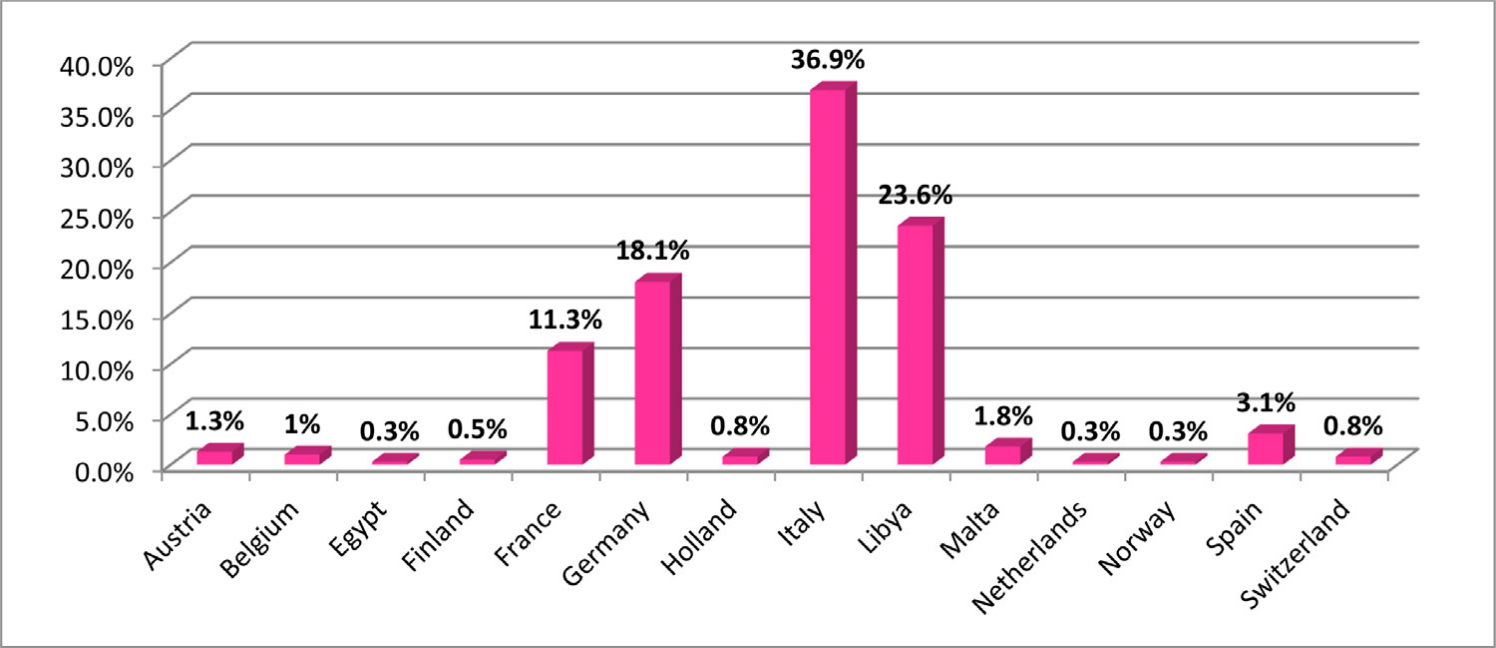
Respondents intended country of destination.

Source: Field Survey, 2018.

**Fig. 2:**
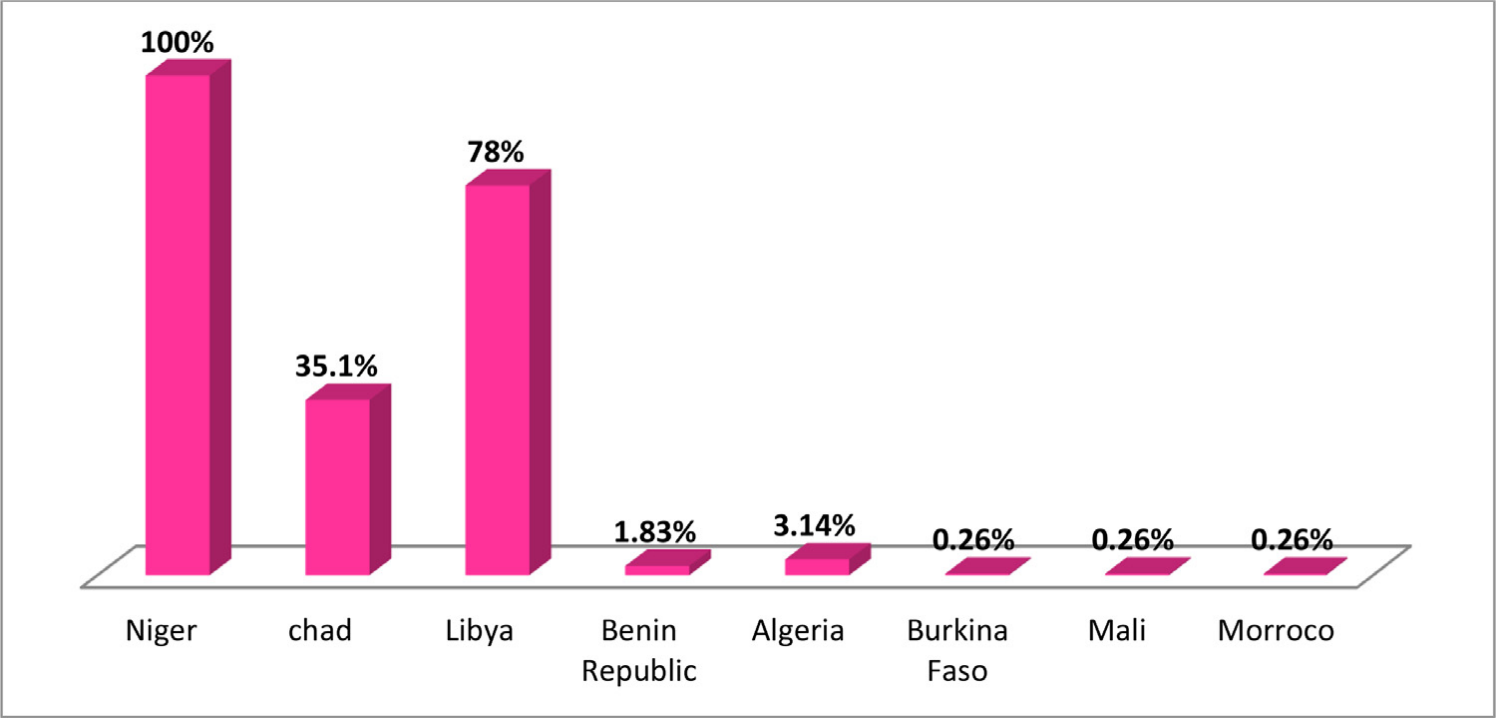
Respondents’ transit countries.

Source: Field Survey, 2018.

In terms of the country in which the respondents intend to migrate for a new living, this study revealed, as shown in Fig. 1, several countries in Europe and few countries in North Africa. Italy was the most frequent, with 36.9% among the respondents, followed by Libya (23.6%). Germany (18.1%) and France (11.3%) also recorded high response rate among the respondents. As revealed in the study, the road was the main route of migration by all the respondents. Among 76.1% of the respondents who had the intention to cross through the Mediterranean sea, 52.5% of them were those who have already entered the Mediterranean Sea but were either stranded or caught on the sea and consequently could not cross to Europe or concluded their journey to their intended country of destination (Fig. 2).

### Transit countries

Niger was a major route and transit country for illegal migration. All the respondents passed through and stopover in Niger. The major stopover cities in Niger were Agadez and Zinder along the desert area. Another major transit country was Chad 134(35.1%), as some passed from Niger to Chad and then entered Libya (Fig. 2). Libya was the major transit for those destined for countries in Europe. Most of the respondents 368(96.3%) do not possess legal travel documents while crossed the borders. The majority of them were smuggled 374(97.9%) and trafficked 369(96.6%) into other countries during the migration. Those smuggled were migrants of whom their network recruiters illegally facilitated their entry into another country, while those trafficked were exploited by traffickers for the purpose of force labour or commercial exploitation. However, as respondents revealed, most of those smuggled from one country to another were also trafficked at some point during their migration.

## Drug use among migrants

### Respondents that have used drugs during their migration

The study showed that 234(61.3%) of the respondents have used drugs during their migration. Drug use was predominant among those in the younger age group (26 – 30) accounting for 24.9%. Among male respondents, more than three quarter 170(71%) of them used drugs, while about 64(44%) among female respondents also used drugs. Of the total 234 respondents (61.3%) who reported to have engaged in drug use during migration, about 35(15%) of them were reported to have been engaged in drug use before leaving the shores of Nigeria.

### Reason for drug use during migration

The respondents when asked the reason for their drug use during migration, among the 234 respondents that have used drugs, ‘frustration’ 189(49.5%) was the leading cause of their drug use. This was followed by their being ‘stranded’ 109(28.5%) and ‘trauma’ accounting for 95(24.9%) among the respondents. One respondent stated, *“I lost my two brothers inside the sea and because of that I take drugs to forget thinking, and also I was jailed in prison”* (IDI-10, male respondent). Few of the respondents revealed that eagerness to continue the journey (9.4%), peer pressure 25(6.5%), no employment in the country of migration 14(3.7%) and introduction to drug business 13(3.4%) as factors that led to their drug use. Some respondents who shared their experiences stated that they use drugs to make them feel less hungry, while some were compelled to use these drugs as the following statement reflect: *“In Libya, those who kidnapped us also give us these drugs, so that we will do whatever they ask us to do”* (FGD-09, male respondent). Some of the respondents shared their experiences regarding taken these drugs under compulsion, as one respondent explained, *“The Libyans forced me to take drugs. I was selected to take care of my fellow returnees and for this purpose they use to give us drugs to make us aggressive”* (IDI-03, female respondent).

Access to drugs was noted to have also contributed to the reason for the drug use among the respondents, while some of these drugs were sold in the camp or prisons in Libya. Examples of these drugs include marijuana, hashish, shisha and tramadol.

### Types of drugs used during migration

**Fig. 3:**
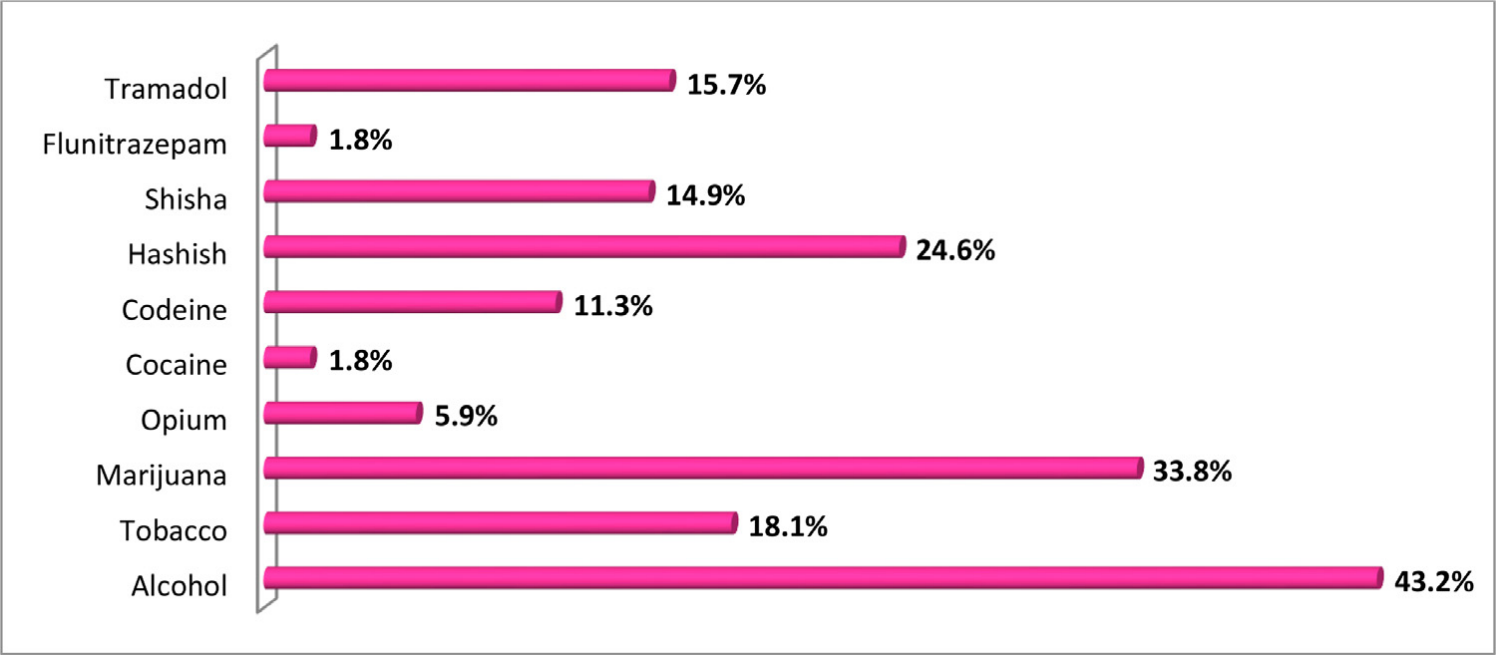
Type of drugs used by the respondents during migration.

Source: Field Survey, 2018.

In terms of drug-types used during migration as shown in Fig. 3, the largest number of the respondents indicated the use of alcohol (43.2%), followed by marijuana (33.8%) and hashish (24.6%). Tobacco (cigarette) use was 18.1%, Tramadol use 15.7%, shisha (14.9%) and codeine accounted for 11.3%. Other drug-types found in used by the respondents were opium (5.9%), cocaine (1.8%) and flunitrazepam (1.8%).

### Illegal activities engaged in by the respondents during migration and are linked to drug use

Respondents were asked whether they got involved in any illegal activities or crime in the country of migration, and if the crime is related to drug use. In their responses, the illegal activities or crime engaged by the respondents during migration were drug business (50.8%), commercial sex work and stealing, 40.7% and 8.5%, respectively. Likewise, some migrants in cooperation with the Libyans were involved in abduction of other migrants and negotiation of ransom. Most illegal business actitivies were performed in Libya by the Nigerian migrants. While some engaged in these activities as a means of survival, some were forced into the acts through trafficking. Among the respondents involved in illegal business activities or crime during migration, 22% of the respondents’ illegal businesses have a link to drug use. On further exploration, the respondents talk about drugs being given to them by the traffickers before embarking on stealing or prostitution. The drug business led some into drug use while possessing the drugs.

### Respondents’ drug use and problematic drug users

Table 2 showed the prevalence of drug use among the migrant returnees in the survey. Of the 234 (61.3%) who use drugs, 124 (52.99%) responded that they became problematic drug users or drug dependent. And only 38 (30.64%) of them seek for help in Libya. A respondent said, *“My friend introduced me to drugs in Niger during our journey. I was addicted to these drugs, particularly shisha, because if I don't take it I will not be okay, whenever I take it I will be calm and will be okay. It was not easy for me to stop drugs, because I was actually getting used to it”* (IDI-07, male respondent) Table 3.

**Table 2:** Respondents drug use and problematic drug use.

	Yes		No		Total No. of Respondents (n)
	n	%	n	%	
Respondents that use drugs	234	61.30	148	38.70	382
Respondents with problematic drug use	124	52.99	110	47.01	234
Respondents with problematic drug use who sought for help in Libya	38	30.64	86	69.35	124

**Table 3:** Kind of help support in Libya.

Where Respondent Seek Help	No. of Respondents (n)	Kind of Help & No. of Respondents (n)
Friend	25	Talk to a friend (25)
NGO	3	Rehab (1)
		counseling (1)
		Treatment (1)
Faith-Based(church)	3	Rehab (3)
Health Clinic	7	Treatment (7)
Total	38	38

On further probe, the respondents were of the views that help for problematic drug use or treatment seeking could not be easy for the irregular migrants and most were held hostage in the transit countries. In Libya, the respondents who became problematic drug users, 6.45% seek for help in a treatment center (health clinic or Nongovernmental Organisation [NGO]), 3.23% seek for rehabilitation in NGO or faith-based centre, while 0.81% seeks for counselling in NGO. Other respondents (20.15%) only discuss their drug use disorder with their friends.

### Respondents who use drugs on their return to Nigeria

The study also depicts that among the total respondents (382) in the survey, 147 (38.5%) used drugs on their return to Nigeria. This result, when compared to percentage that used drugs in immigration, 234(61.3%) used drugs during their migration while 147(38.5%) used drugs on their return to Nigeria. The respondents when asked the reason for drug use when they returned to Nigeria, 80 (30.4%) responded that they were already dependent on drugs, 76 (28.90%) responded that they were frustrated on their return to Nigeria, 63 (24%) responded that they resort to drug use due to lack of employment, and 44 (16.7%) alleged that pre and post-migration stress and trauma contributed to their drug use problem in Nigeria.

### Drugs used by the respondents on their return to Nigeria

**Fig. 4:**
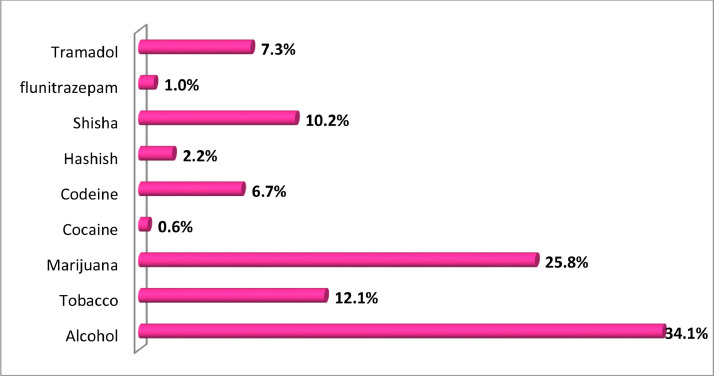
Drugs used by respondents on their return to Nigeria.

Source: Field Survey, 2018.

The drugs used by the respondents on their return to Nigeria where alcohol (34.1%), marijuana (25.8%), cigarette tobacco (12.1%), shisha (10.2%), tramadol (7.3%), codeine (6.7%), hashish (2.2%), flunitrazepam (1.0%) and cocaine (0.6%). Alcohol and marijuana abuse were most dominant among the respondents (Fig. 4).

### Respondents that continued drug use after undergoing reintegration programme or after three months reunion with their families in Nigeria

Of the 147 (38.5%) among the study population that uses drugs on their return to Nigeria, 136 (93%) continued to use drugs after undergoing reintegration programme or after 3 months reunion with their family. One respondent stated, *“I got addicted to drugs. There are times if I have not taken these drugs, it will look as if I am shivering, but I hide it from my parents when I came back to Nigeria, I do go to buy these drug such as tramadol and marijuana, and once I take it, I will just take tom-tom (candy)”* (IDI-05, female respondent).

The qualitative study of this survey revealed that the reintegration programmes undergone by the respondents were mainly on business and entrepreneurship skill training and empowerment programmes in the aspect of business establishment for the migrant returnees. The training comprised enterprise development modules such as how to generate business ideas, how to start your business, how to develop a business plan, record keeping, and business partnership and cooperatives. None of these programmes has a psychosocial and behavioural therapy component that addresses the issue of drugs and addiction among the migrant returnees. The result of this study shows that the current reintegration programme and family reunion has not expressively reduced drug use among these migrant returnees.

In relation to the reason for the migrant continued drug use after their reintegration programme or three months family reunion, 59 (43.4%) were still dependent on drugs, 31 (22.8%) were still frustrated, 28 (20.6%) were still passing through pre and post-migration stress and trauma, and 18 (13.2%) still use drugs as a result of no employment. The respondents 58(39.7%) who use drugs complained to have other medical effects associated with their drug use, such as acute cough, amnesia, chest pain, constant fever, headache, high blood pressure, internal pain, stomach pain, throat pain, itching and mental challenge.

### Respondents who have been involved in treatment programme specifically related to drug use in Nigeria

The study shows that most respondents 125(85.3%) that use drugs did not seek treatment since their return to Nigeria. Of those who did seek treatment, 22(14.7%) were males. None of the females seek for treatment upon their return in Nigeria. Stigmatization remains one of the reasons for none-treatment seeking among the migrant returnees.

### Stigmatization and discrimination face by the migrant returnees upon their return and as a result of their drug use

The respondents in this study complained that they have faced stigmatization and discrimination upon their return to Nigeria. Apart from the stereotype stigma that have been placed on migrant returnees from Libya who are associated with irregular migration and human trafficking, the migrants’ drug use problem also increases the societal stigma and discrimination encountered by these population as the following statements illustrate: *“Some of our girls that travel to Libya and came back, if people knew you came back from Libya, it is big stigma in Edo state, they feel maybe you have smoked or taken the whole drugs in Libya and they see us as abnormal human beings”(*FGD-19*,* female respondents); *“Because of the stigma, most of us that came back from Libya, our smoking and drug taking increased, immediately we came back, with countless interviews, facing cameras. With your face on TV, your friend may call his family that they saw you and that you are back to the country from Libya, walking on the streets, some people start murmuring against you, that he is a Libyan returnee, he has been involved in crime in Libya, these are stigma, and it makes one go crazy”* (FGD-05 – male respondents).

## Drug trafficking among migrants

### Respondents’ engagement in illicit drug peddling or trafficking in country of migration

Of the total respondents of 382, 60 (15.7%) disclosed that they trafficked drug during their migration (in Libya or other transit countries) and among them were 40 (10.5%) who also carry these drugs on their way through the journey. On further probes, the respondents revealed that the majors ways of doing this is to boycott border checks through alternative (secret) route or to hide it in a secret place in the migrant luggage, cloths, underwear or food. Some of the respondents disclosed that they kept the drugs in their body by swallowing them while others ‘settle’ by bribing the officers on duty. Among the total female respondents (144) in the study, 4% of them were reported to be involved in drug trafficking. The information gathered from FGD further revealed that pregnant women among migrants are also used in the drug trafficking.

### Drugs trafficked or peddled by respondents during migration

**Fig. 5:**
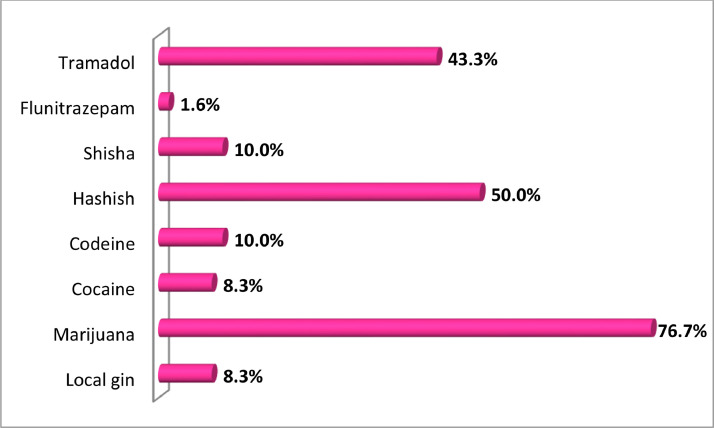
Drugs trafficked or peddled by migrant during migration.

Source: Field Survey, 2018.

The respondents that attested to their involvement in either drug business, mentioned the following drugs as the major drug with highest drug trafficked volume or that move sales of which they were involved, and these were: Marijuana (76.7%), Hashish (50%) and Tramadol (43.3%). Other drugs trafficked or peddled were listed in Fig. 5. The local gin (spirit) observed in this study were those not under normal government regulation. This illicit gin has been reported to be toxic and banned in some countries such as in Nigeria by the National Agency for Food and Drug Administration (NAFDAC).

### Groups or persons responsible for respondents’ introduction to drug business

Among groups or persons through whom respondents were introduced to drug business, findings shows that the highest influence on the migrants towards drug business were through migrant smugglers (46.7%), followed by human traffickers (45%) and friends (25%). Some of the respondents also disclosed through in-depth interviews and focus group discussions that their family members introduced them to drug business. Further investigations revealed that some respondents in this category got into drug business in Libya or transits countries due to survival and frustration experience.

The respondents also claimed compulsion by some Libyan rebels or officers in their prisons or refugee camp to sell drugs for them, while some of migrants were people that started drug business before leaving Nigeria. The reasons for being involved in illicit drug business during migration, as mentioned by the respondents who were directly involved in drug business, the most reason stated was to raise money (33%) for their journey. This was followed by compulsion or being forced into drug business (32%), as this was supported by information obtained from the focused group discussion in which many of the migrant claimed that their camp officers and human traffickers put them under threats and compulsion to the drug business. Other major reasons for the respondents’ engagement in dug business were frustration (3%) and solely for survival (i.e. to earn a living) (15%). The other remaining respondents (17%) involved in the drug business did not give any tangible reasons for their involvement in drug business. The following statements illustrate: “*Some of this human traffickers will approach you that they can help you go to Europe, they will charge you 700,000 Naira and you will pay them, but once you get to Agadez in Niger, there will be a disconnect, these traffickers phone numbers will be switched off, then other traffickers will come in and approach you to carry drugs for them, since you don't have money with you, you will agree to do it, poverty, frustration are responsible for drug trafficking, sometimes this human traffickers force it on you”* (FGD-02, male respondents); *“I deal on drugs, I sell drug to gather money, to travel to Libya, when I got to Libya I continued selling drugs to survive, even after returning to Nigeria, I still deal on drugs even now, just for me to survive”* (FGD-08, male respondents).

### Respondents arrested for possession of illegal drugs during their migration

In the study population of 60 migrant returnees involved in drug business, only 28% of them have had been arrested or detained by law enforcement agent (in Libya) due to their illegal action on drug business. Among this population, 28% have had experience of arrest, 12% were females. Information gathered from the focus group discussions revealed that some of the irregular migrants involved in drug business bribe some law enforcement agents to escape legal prosecution.

## Discussion

Irregular migration along the Central Mediterranean route is increasingly dangerous for migrants (International Organization for Migration 2016a). This study revealed the prevalence of drug use among migrant returnees. Irregular migrants, according to Fazel, Wheeler, and Danesh, usually witnessed or personally experienced pre and post-migration stress and trauma, including experience of frustration (Fazel et al., 2005), as revealed in the findings of this study. As such, the above factors make them vulnerable to drugs use, and it is no surprising that the prevalence of drug use is high among this population. The challenges and other process of psychological change that follow the experience in irregular migration may lead migrants to have engaged in drug use (Berry, 1997). In this study, drug use was predominant among the age group 26 – 30, accounting for 24.9% among the respondents. The study also depicts high percentage (89.51%) of non-treatment seekers among migrants who are problematic drug users in the country of migration.

There is a dearth of literature on irregular migrants and drug use, precisely in Africa, but a growing body of research, predominantly conducted among vulnerable populations of those with problematic drug use has found non-treatment seeking to be high among this population. According to the 2014 United States National Survey on Drug Use and Health, people may be reluctant to seek treatment for drug use because of denial of their drug use disorder, societal stigma, and time constraints (Substance Abuse and Mental Health Services Administration 2019). In United States, about 85% of those with problematic drug use have not received treatment. In this study, the illegal status of the respondents also poses a challenge in seeking treatment in the country, of which they do not have legal position. The present reintegration programmes or the family reunion has not contributed to reduction in drug use among the migrant returnees. Of the 147 (38.5%) respondents among the study population that use drugs on their return to Nigeria, 136 (93%) continue to use drugs after undergoing reintegration programmes or after three months reunion with their family. Only 11 (7%) have stopped using drugs. It was observed that the present reintegration programmes by government, non-governmental, intergovernmental and international agencies that have been provided for these migrant returnees lacks psychosocial and behavioural therapy component that addresses the issue of drugs and addiction among the migrant returnees. Rather, the reintegration was mainly on business and entrepreneurship skill training and economic empowerment.

Although, some studies have established that employment can reduce the likelihood of drug use or aid recovery (Montoya, 2004). It is also believed that the family also plays a role in the recovery process (Substance Abuse and mental Health Administration 2015). The result of this study showed that the current reintegration program or family reunion has not reduced drug use among these migrant returnees. Research, as conducted by Anikeeva et al. (2010) and Gushulak et al. (2011)*,* found that migrant health decreases over time to a range of factors, including reintegration challenges and barriers to health service use, which emphasizes the importance of maintaining contact with arrived migrants to monitor changes in drug use during the early post-migration period. Migrants, particularly those who have passed through ill-treatment and abuse, commonly experience social and economic inequality, marginalization and discrimination (Fozdar and Hartley, 2014, Capps et al., 2015). Many of the migrant returnees in this study reported being stigmatized and discriminated against; factors that are important determinants of health (Frohlich and Potvin, 2008, Steel et al., 2009), and may contribute to feelings of stress and hopelessness which may, in turn, contribute to drug use problem. For instance, the result of this study showed that 85.3% of the migrant returnees have not been involved in any treatment programme since their return to Nigeria. However, 14.7% that seek treatment were male. Societal stigma, among other factors, could contribute to non-treatment seeking upon return of these migrants. Stigma has also been expressed as a factor in non-treatment seeking by studies of Gorman (2014), Appel et al. (2004), and Mojtabai et al. (2014).

The Central Mediterranean route, through the Sahara desert, has been observed in this study as drug trafficking route. From the result of the analysis, the migrant returnees get into drug trafficking/peddling through the activities of the migrant smugglers, transit countries citizens and human traffickers which was also reported by Simon (2017). Similar findings was also observed in the study of Slack and Whiteford^21^ and Triandafyllidou et al. (2012), who associate migrant smuggling networks with illicit activities such as kidnapping and migrant participation in drug trafficking. However, the above authors also highlighted the migrants’ agency and noted that these networks do not resemble Mafia organizations. Migrant returnees most time get involved in trafficking or peddling as well as other related crimes during their migration, as this was done for surviving during the journey. Specifically, it was done to raise money to complete their irregular migration as some get into drug trafficking due to compulsion by human traffickers, migrant smugglers or citizens of transit countries. This is in line with the findings of Simon in his work titled ‘From victims of trafficking to felons: Migrant smugglers recruited by Mexican cartels’ (Simon, 2017).

The current study compliments the findings of past researchers who worked on UNODC research paper titled ‘The role of organized crime in the smuggling of migrant from west Africa to the European Union (UNODC 2011), as they concluded that fostering of socio-economic development (such as vocational business training) in the countries experiencing irregular migration such as Nigeria would help to further reduce demand for smuggling service. This study has identified that migrant returnees are presently facing employment challenges which is the main reasons why they are illegally migrating to other countries and most being involved in drug trafficking, and failure to secure jobs can keep up the trend of further irregular migration, drug peddling and other crimes among these return migrants if care is not taken.

In summary, Alcohol (43.2%), marijuana (33.8%) and hashish (24.6%) constituted most of the drugs used by respondents on immigration. The percentage that used drugs in immigration was 61.3%, and 38.5% used drugs on their return to Nigeria. Considering the prevalence of 14.4% drug use in Nigerian based on 2017 National Household Survey (UNODC 2018). Drugs mostly trafficked were Marijuana (76.7%), Hashish (50%) and Tramadol (43.3%). The study revealed that 15.7% of the respondents engaged in drug trafficking during their migration, and 28% of the respondents that trafficked drugs had previous experience of arrest or detention by law enforcement agent.

It is recommended that providing access to treatment and addressing the underlying factors which lead to drug use is essential. Therefore, evidence-based responses to drug use should be provided to returned migrants on immediate return to include health screening, psychosocial support and medication-assisted therapies for drug use dependence and withdrawal. It will be very essential for drug use services to be integrated with mental health services for trauma-informed care. The existing reintegration programmes for the migrant returnees should be reviewed to incorporate psychosocial and behavioural therapy component that addresses drug use problem and involvement of their families to ensure sustainable reintegration. Sensitization and advocacy at the grassroots level involving communities and schools would help in creating awareness and educate most especially the young people on the dangers of irregular migration and human trafficking, with emphasis on predisposing factors leading to drug use and penalties for the offence on drug trafficking. The emphasis is to promote regular migration. Supporting legal entry into any country ensures safety, worthy livelihood and entitled health benefits or coverage to migrants. Priority should focus on improving border securities and cooperation with member states and international border control to reduce security threat and corruption, and adopting sustainable measures to amend the lapses in border checks such as bribery and smuggling.

Disclosure was a challenge observed among the female respondents. Most female respondents were reluctant to disclose their migration experience, most especially those involved in prostitution. Perhaps the trauma or stigma could be the reason and owing to the sensitive nature of this research. Efforts were made to generate pool of data from the female using a female interviewer in most of the difficult encounters.

## Conclusions

The prevalence of drug use in irregular migration among the study population was 61.3% which draws attention to the need to understand the epidemiology of drug use among irregular migrant populations, particularly among persons who fall victim to deception, coercion, human trafficking and migrant smuggling. Experience of migration stress, trauma and frustration were among the factors that contribute to drug use among the study population. Non-treatment seeking, both in the migration countries and in the return to Nigeria, was high among those with problematic drug use. The existing migrant returnees’ reintegration programmes lack components of psychosocial and behavioural therapy that address drug use problem. However, stereotype stigma as a result of involvement in irregular migration and drug use has pose negative effects among this community. The study also discovered that some of the migrants get into drug business primarily to raise money for survival, while some were compelled into the business. The major influencers through whom the migrants were introduced to drugs were the migrant smugglers, human traffickers and friends within the migrants’ social group. On this basis, there is need to draw attention of the findings, with important implications for public health and social reintegration.

## Declaration of Competing Interest

The authors declare that they have no known competing financial interests or personal relationships that could have appeared to influence the work reported in this paper.
